# Healthcare access and socio-demographic determinants of estimated 10-year risk of cardiovascular diseases in Indonesia: A population-based study

**DOI:** 10.1371/journal.pone.0318112

**Published:** 2025-08-20

**Authors:** Sujarwoto Sujarwoto, Asri Maharani, Devarsetty Praveen, Anna Palagyi, Prem S. G. Kumar, Seye Abimbola, Gindo Tampubolon, Anushka Patel

**Affiliations:** 1 Department of Public Administration, University of Brawijaya, Malang, Indonesia; 2 Division of Nursing, Midwifery and Social Work, School of Health Sciences, University of Manchester, Manchester Academic Health Science Centre (MAHSC), Manchester, United Kingdom; 3 The George Institute for Global Health, India, Hyderabad, India; 4 The George Institute for Global Health, University of New South Wales, Sydney, Australia; 5 School of Public Health, University of Sydney, Sydney, Australia; 6 Global Development Institute, University of Manchester, Manchester, United Kingdom; Bangladesh University of Health Sciences, BANGLADESH

## Abstract

**Background:**

Cardiovascular diseases (CVDs) are the leading cause of morbidity and mortality in Indonesia. Despite the importance of identifying individuals at high risk of CVDs for Indonesian health planners in designing effective intervention strategies, the CVD situation in the country has not been well-documented. This study aimed to estimate the distribution of the estimated 10-year risk of CVD and the associated socio-demographic factors and healthcare access in Indonesia.

**Methods:**

This study was a community-based study in which the data were collected using interviews and the taking of physical measurements of 903,130 adults aged 40 years and older in 390 villages in Malang District, East Java Province, Indonesia, from January 2020 to February 2024. The estimated 10-year risk of CVD was calculated based on the World Health Organization/International Society of Hypertension’s region-specific charts for the Southeast Asia Region (SEAR B). We performed multilevel logistic regression modelling to examine the associations between individual and healthcare provider densities and the estimated 10-year risk of CVD, as well as receiving optimal preventive treatment, defined as at least one blood pressure-lowering drug and a statin for all high-risk individuals, and an antiplatelet drug for those with prior diagnosed CVD.

**Results:**

Among 903,130 participants, 169,758 (18.8%; 95% Confidence Intervals 18.7% − 18.9%) had high cardiovascular risk. The proportion of high CVD risk was greater (<0.001) in urban (19.6%) than in rural areas (18.3%). Only 25.7% of all the respondents with high CVD risk received optimal preventive treatment, with high-risk males who live in urban areas showing better treatment. The availability of community-based health care (*Posbindu*), medical doctor at primary healthcare, nurses, and health insurance were associated with lower odds of having high CVD risk.

**Conclusion:**

Around one-fifth of the population aged 40+ in Malang District, Indonesia is estimated to have high 10-year CVD risks, as assessed by the WHO/ISH risk prediction charts, and three-quarters of those with high risk did not receive optimal preventive treatment. Ensuring that individuals with high CVD risk get the optimal treatment is important, especially in low- and middle-income countries. The accessibility of preventive care is vital in primary care to address the sex and geographical gap of CVD risk management.

## Introduction

The epidemic of cardiovascular diseases (CVDs) has shifted from high-income to low- and middle-income countries. Approximately 80% of CVD deaths occurred in low-income countries in 2019 [1,2]. Indonesia – reaching upper-middle-income status since 2023 – is no different, where an estimated 5.3% of the population had CVDs in 2019. These diseases are the country’s leading cause of morbidity and mortality, responsible for more than a third (38%) of all deaths. Years of life lost due to premature mortality from CVDs in 2019 are estimated to be 5,826 years of life lost/100,000 [1].

Studies have shown that the high burden of CVDs in Indonesia is attributable to preventable vascular risk factors, particularly hypertension, obesity, and active tobacco use [3–5]. Our prior studies further found a high unmet need for CVD care [6]. Data from eight villages in Malang in 2016–2017 found that only 11% and 1% of all the respondents with high estimated 10-year CVD risk were on blood pressure lowering and statin treatment [5]. Residents of urban areas were more likely to receive blood pressure lowering and statin treatment than residents of semi-urban and rural areas. Prior nationwide studies also found that individuals with higher per capita expenditure were more likely to have their cardiovascular care needs met [6]. The absence of a universal healthcare insurance scheme is one possible explanation for the demonstrable inequality in cardiovascular care.

The government of Indonesia has launched several national programs to improve CVD risk management in primary health care. This included a national health insurance scheme, the *Jaminan Kesehatan Nasional* (JKN) in 2014 [7,8]. By March 2023, 254.9 million people (92.2%) were already covered by BPJS insurance, both via non-contributory and contributory schemes [9]. In addition to financial protection, the government has included non-communicable disease (NCD) risk screening within a national minimum public services standard since 2014. The standard mandates all primary health care services to deliver NCD risk screening to all citizens 15 and older at least once a year [10]. The Ministry of Health organizes health promotion activities through a community engagement called Pos Pelayanan Terpadu (Posbindu) to support primary healthcare workers. This community engagement focuses on raising public awareness, early screening, and early detection of NCDs by empowering non-voluntary health workers or *kaders* in the communities. Moreover, various local innovative programs to improve cardiovascular risk management in the communities and primary health care have also been raised, such as the implementation of a digital health-supported program for cardiovascular risk screening and management (SMARThealth) in Malang district, East Java since 2019 [11]. However, despite these national and local measures, there is still inadequate information about the distribution of CVDs and the associated factors in Indonesia for further planning of an effective service response.

The aim of this study was to determine the distribution of estimated 10-year CVD risk across communities based on sex and different levels of urbanization and to identify determinants associated with the estimated 10-year CVD risk among population 40 and older in Malang district, East Java province.

## Subjects and methods

The study received ethics approval from the University of the New South Wales Human Research Ethical Approval (HC190531) and the Ethical Committee, Ministry of Research, Technology, and Higher Education, Medical Faculty of Brawijaya University (Reference: 236/EC/KEPK/09/2019).

### Settings and study population

This study was conducted in Malang district of East Java province. Malang is the second-largest district in the province. Its area covers 3,535 square kilometres, with an agricultural emphasis on rice and sugar cane. Malang’s total population is 2,635,950, with 1,240,162 population 40 and older distributed across 33 sub-districts, 390 villages, 244 (62.6%) rural, and 146 (37.4%) urban [12]. Malang has 39 primary health centers or *Pusat Kesehatan Masyarakat* (*Puskesma*s) (1 per ∼65,000 individuals), 390 village health clinics or *Pondok Kesehatan Desa* (*Ponkesdes*) (1 per ∼7,000 individuals) and 4,111 community-based healthcare or *Posbindu* [13]. In 2020, 10.2% of the population in Malang district was ‘poor or near poor’ [13].

**Fig 1:**
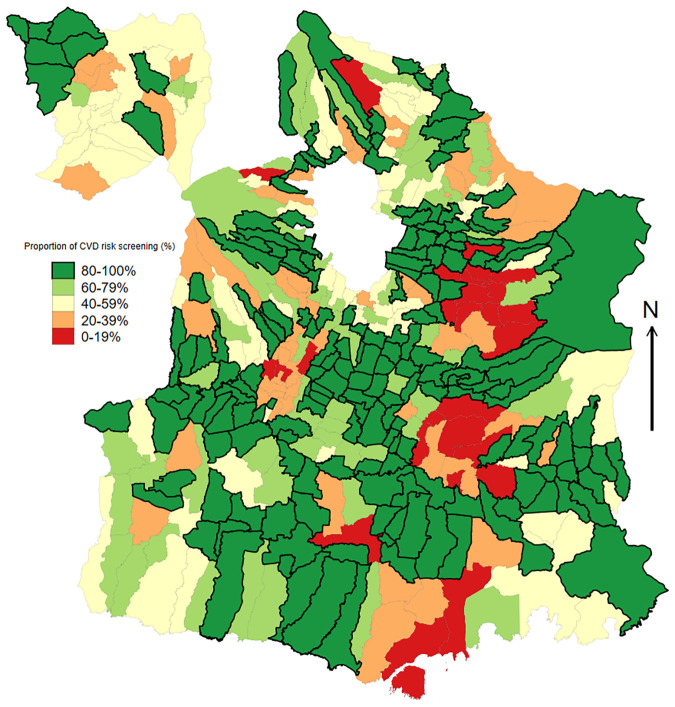
Distribution of cardiovascular disease risk screening among population aged 40 and above across 390 villages in Malang District, East Java between January 2020 and February 2024. The figures comply with the CC BY 4.0 license in which it was created using ArcGIS software using public domain shapefile data from the USGS National Map Viewer.

The current study was conducted among adults 40 years of age and older in all 390 villages of the district of Malang who underwent cardiovascular screening between 1 January 2020 and 29 February 2024. Trained community health workers or *kader* recruited by Malang District Health Office screened 903,130 individuals, representing 72.82% (903,130/1,240,162) of the total population of ≥40 years in the district. Fig 1 describes the geographical distribution of CVD risk screening across 390 villages, which highlights variations in the number of screened individuals across the villages.

### Study design

This study was a community-based study in which the data were administrative data from Malang District Health Office from 1 January 2020–29 February 2024. The data was accessed for research purposes on 15 March 2024. All data were fully anonymized before we accessed them, and we had no access to information that could identify individual participants during or after data collection.

#### Data collection.

The methods for completing the epidemiological questionnaires and anthropometric measurements were based on a standard protocol [14,15]. Data collection was performed by trained health workers who use electronic data records in primary health care (*Puskesmas* and *Ponkesdes*) called electronic *Puskesmas* (ePuskesmas) to collect information on demographic status, socioeconomic status, medical history, family medical history, smoking status, salt and vegetable consumption, physical activity levels, BPJS insurance ownership. For individuals who are not able to visit *Puskesmas* and *Ponkesdes,* data collection was conducted by trained community health workers or *kader* using SMARThealth apps and e-puskesmas for kader app [5,11,16,17]. The data from SMARTheath apps were standardized and recorded in the *Puskesmas* electronic data record so that all individual variables used in this study have similar measurements*.*

The anthropometric measurements included height and weight. Height measurements were accurate to 1 cm, and weight measurements to 1 kg. BMI was calculated as body weight (kg) divided by height squared (m2). Blood pressure and heart rate were measured using a digital sphygmomanometer (OMRON HEM-7130 made in Japan) with an appropriate size cuff following the standard recommended procedures. For the data collected using SMARThealth apps, Kaders took blood pressure readings three times at 5-minute intervals after 5 minutes of rest in the sitting position with positioning of the arm, with the average used for data analysis. Random blood glucose rates were measured using the pin-prick method and portable glucometers (FreeStyle Optium Neo) [14].

Data at the community level was retrieved from the Indonesia village potential census (*Podes*) and official statistics. The *Podes* census is a bi-annual census aimed at collecting the socioeconomic characteristics of Indonesian villages. The census was conducted by the Indonesia National Bureau of Statistics and has been delivered since 1980 [18].

#### 10-year risk for cardiovascular diseases.

The individuals were categorized as having hypertension if they had a self-reported history of hypertension diagnosed by a physician, was on hypertension treatment, or had a systolic blood pressure (SBP) ≥140 mmHg and/or a diastolic blood pressure (DBP) ≥90 mmHg. An individual was considered to have diabetes mellitus if they had a self-reported previous physician diagnosis of diabetes, was being treated with blood glucose-lowering medications, or had a random blood glucose measurement of ≥200 mg/dL. Obesity was defined as a BMI of >30 kg/m2.

We calculated the 10-year risk of CVD using the World Health Organization cardiovascular disease (WHO CVD) risk (non-laboratory-based) charts calibrated for use in Southeast Asia region B (SEAR B) [19]. The chart has also been used as a national standard for calculating CVD risk in Indonesia. This calculation included information on age, sex, smoking status, blood pressure, and body mass index (BMI). We used WHO CVD risk non-laboratory-based charts due to the high missing data in random blood glucose (77.5% - please see Table 1). The estimated 10-year CVD risks were categorized into <5%, 5% to <10%, 10% to <20%, 20% to <30%, and ≥30%.

**Table 1:** Socio-demographics and healthcare characteristics of individuals based on sex and rurality.

Variables	All (n = 903,130)	Female (n = 518,922)	Male (n = 384,208)	p-value*	Urban (n = 367,151)	Rural (n = 535,979)	p-value*	Missing data**
Population 40 + , frequency	1,240,162							
Population, frequency	2,756,321							
*Individual-level variables*								
Age (years), mean (SD)	55.6 (11.4)	55.2 (11.3)	56.0 (11.4)	<0.001	54.9 (10.9)	56.0 (11.6)	<0.001	0 (0)
Age group, frequency (%)								
40–44	174,439 (19.3)	103,989 (20.0)	70,450 (18.3)	<0.001	73,262 (20.0)	101,177 (18.9)	<0.001	0 (0)
45–49	142,934 (15.8)	83,716 (16.1)	59,218 (15.4)		61,997 (16.9)	80,937 (15.1)		
50–54	149,124 (16.5)	86,803 (16.7)	62,321 (16.2)		61,543 (16.8)	87,581 (16.3)		
55–59	131,895 (14.6)	76,593 (14.8)	55,302 (14.4)		54,364 (14.8)	77,531 (14.5)		
60–64	111,387 (12.3)	62,586 (12.1)	48,801 (12.7)		44,383 (12.1)	67,004 (12.5)		
65–69	79,521 (8.8)	42,751 (8.2)	36,770 (9.6)		31,066 (8.5)	48,455 (9.0)		
70+	113,830 (12.6)	62,484 (12.0)	51,346 (13.4)		40,536 (11.0)	73,294 (13.7)		
Education level, frequency (%)								0 (0)
Not attending school	66,931 (7.4)	43,832 (8.4)	23,099 (6.0)	<0.001	25,215 (6.9)	41,716 (7.8)	<0.001	
Elementary school	251,128 (27.8)	152,752 (29.4)	98,376 (25.6)		89,769 (24.5)	161,359 (30.1)		
Junior secondary school	465,410 (51.5)	261,655 (50.4)	203,755 (53.0)		184,380 (50.2)	281,030 (52.4)		
High school	89,579 (9.9)	45,985 (8.9)	43,594 (11.3)		50,869 (13.9)	38,710 (7.2)		
University	30,082 (3.3)	14,698 (2.8)	15,384 (4.0)		16,918 (4.6)	13,164 (2.5)		
Marital status, frequency (%)								0 (0)
Married	743,276 (82.3)	413,739 (79.7)	329,537 (85.8)	<0.001	301,043 (82.0)	442,233 (82.5)	<0.001	
Single	79,899 (8.8)	47,056 (9.1)	32,843 (8.5)		31,162 (8.5)	48,737 (9.1)		
Divorced	31,013 (3.4)	20,213 (3.9)	10,800 (2.8)		13,636 (3.7)	17,377 (3.2)		
Widowed	48,942 (5.4)	37,914 (7.3)	11,028 (2.9)		21,310 (5.8)	27,632 (5.2)		
Employment status, frequency (%)								0 (0)
Unemployed	65,482 (7.3)	42047 (8.1)	23,435 (6.1)	<0.001	28,230 (7.7)	37,252 (7.0)	<0.001	
Formal	78,494 (8.7)	27,679 (5.3)	50,815 (13.2)		44,011 (12.0)	34,483 (6.4)		
Informal	555,230 (61.5)	291,146 (56.1)	264,084 (68.7)		201,305 (54.8)	353,925 (66.0)		
Homemaker	138,140 (15.3)	136,046 (26.2)	136,046 (0.5)		66,083 (18.0)	72,057 (13.4)		
Retired	8,220 (0.9)	2,326 (0.4)	5,894 (1.5)		5,263 (1.4)	2,957 (0.6)		
Self-employed	57,564 (6.4)	19,678 (3.8)	37,886 (9.9)		22,259 (6.1)	35,305 (6.6)		
Have BPJS insurance, frequency (%)	510,791 (56.6)	292,219 (56.3)	218,572 (56.9)	<0.001	205,427 (56.0)	305,364 (57.0)	<0.001	0 (0)
*Village-level variables*								
Medical doctor per 1,000 population, mean (SD)	0.1 (0.2)				0.2 (0.3)	0.06 (0.2)	<0.001	0 (0)
*Posbindu* per 1,000 population aged 40 + , mean (SD)	18.4 (3.3)				18.0 (3.2)	18.6 (3.0)	<0.001	0 (0)
Registered pharmacy per 1,000 population, mean (SD)	0.1 (0.1)				0.2 (0.2)	0.02 (0.07)	<0.001	0 (0)
Nurses per 1,000 population, mean (SD)	1.8 (1.6)				1.5 (1.8)	2.9 (1.8)	<0.001	0 (0)
Have an easy access to *Puskesmas*, frequency (%)	388 (99.5)				146 (100.0)	244 (98.0)	<0.001	0 (0)

SD = standard deviation. * Univariable analyses using Kruskal-Wallis test for continuous variables and chi-square tests for categorical variables. ** Presented are n (%)

We further defined high estimated 10-year risk of CVD as the presence of any of the following: (1) a history of CVD confirmed by a physician, (2) an extreme blood pressure elevation (SBP ≥ 160 mmHg or DBP ≥ 100 mmHg, (3) a 10-year estimated CVD risk of 20% or more, or (4) a 10-year estimated CVD risk < 20% and SBP ≥ 140 mmHg and/or DBP ≥ 90 mmHg.

The information on medication was gathered from the primary care medical doctor’s prescription record of blood pressure-lowering drugs, statins and antiplatelet agents. We defined individuals with a high estimated 10-year risk of CVD as receiving optimal preventive treatment if they reported having at least one blood pressure-lowering drug and a statin for all high-risk individuals, and an antiplatelet drug for those with prior diagnosed CVD.

#### Risk factors of high 10-year cardiovascular risks.

We included the risk factors of the estimated 10-year CVD risk at individual and village levels. Determinants at the individual level consist of marital status, educational attainment, employment status, salt and vegetable consumption, physical activity levels, BPJS insurance enrolment and urban/rural domicile. We categorized marital status as married (reference), single, divorced and widowed. Educational attainment was classified as no education (reference), primary school, junior secondary school, high secondary school, and university. Employment status has six categories, i.e., unemployed (reference), formal, informal, homemaker, retired and self-employed. Informal workers are those working on an irregular or flexible basis, often to meet a fluctuating demand for work. The information on health behaviour was based on self-reported having a high salt intake (> 1 tablespoon/day), fruit and vegetable consumption less than 5 portions per day, and physical activity less than 3 times/week.

The information on village-level determinants was retrieved from *Podes* 2022. We included data on the number of community-based healthcare providers for NCD prevention (*Posbindu*), doctors and nurses, registered pharmacies, and access to the nearest primary healthcare provider or *Puskesmas.* We retrieved the total number of populations ≥40 and older in each village and access to the nearest primary healthcare from district official statistics. Then, we calculated the number of doctors, nurses and registered pharmacies per 1,000 population and the number of *Posbindu* per 1,000 population 40 and older (Supplementary file S1 Table provides detailed information about each variable used for statistical analysis). Regarding the healthcare access, we categorized the villages into having an easy access to Puskesmas or not.

### Statistical analyses

We presented continuous variables as a mean ± standard deviation and categorical variables as frequency and percent. Continuous variables between different sex and rurality were compared using Kruskal-Wallis test, while categorical variables were compared using chi-square tests. We provided the proportion of the high estimated 10-year CVD risks to capture the differences in risk factors for males and females. The risk factors were further examined by rurality to highlight the geographic and socio-environmental differences that influence health outcomes. We classified marital status, type of job, educational level, salt and vegetable intake, physical activity, BPJS insurance status, and years of cardiovascular risk screening, according to sex and rurality status.

This study used multilevel logistic regression models to explore the association between individual and village characteristics with a high estimated 10-year CVD risk. These models allow us to address higher-level, i.e., village heterogeneity, assuming that the association between the dependent variable and its covariates varies between villages and individual levels [20,21]. Hence, the models account for clustering individuals in villages by separating individual variance in CVD risks from village variance. The links between variations of village healthcare characteristics and high estimated 10-year CVD risk among individuals can thus be examined appropriately. We follow prior epidemiological studies, which used this model to examine contextual determinants of health [22,23].

The multilevel logistic regression analyses used three models. The first model is the univariable analysis, which includes only each risk factor separately to get the crude odds ratio. Models 2 and 3 are multivariable analyses, as we included several risk factors in one model. We included all the individual-level variables in the second model and the density of the healthcare providers as the village-level determinants in the final model.

We further calculated the proportions and 95% confidence intervals (CIs) of individuals with a high estimated 10-year CVD risk receiving treatment. The numerator was the number of those receiving treatment, and the denominator was the number of those with high cardiovascular risk.

Given that 77.5% of participants had missing random blood glucose values, we followed WHO’s recommendation to use non-laboratory-based charts when biomarker data are unreliable or unavailable. This approach ensures that our risk estimation is unbiased and representative, avoiding selection bias that could arise from excluding a large proportion of participants due to missing blood glucose data. For other variables, missing data were minimal (<0.08%), and we used a complete case analysis approach. Given the large dataset (n = 903,130), this approach was deemed appropriate to maintain consistency across models without substantial loss of power. We present odd ratios (OR), *p-*value, CIs, individual level and village level variance in high estimated 10-year CVD risk, intraclass correlation (ICC), and the −2 Loglikelihood, which serves as an indicator of model fit. All statistical analyses were performed using Stata 18.0.

## Results

### Univariate and univariable analysis

Table 1 illustrates the descriptive statistics of 903,130 individuals and compares their characteristics according to sex and rurality. Most respondents were female (57.4%) and lived in rural areas (59.3%). The mean age of respondents was 55.6 (±11.4) years, with 19.3% aged 40–44 years. Most individuals were educated in junior secondary school or lower (86.7%) and married (82.3%). Over half of the individuals worked as informal workers (61.5%). Around 56.6% of the individuals were enrolled on BPJS insurance. On average, each village had 0.1 (SD = 0.2), doctors, 0.1 (SD = 0.1) registered pharmacies and 1.8 (SD = 1.6) nurses per 1,000 population. The average density of *Posbindu* per population aged 40 + was 18.4 (SD (3.3). The univariable analyses further show that males were likely to be older, had higher education attainment, married and had BPJS insurance (p < 0.001). Urban areas tend to have a higher density of doctors, Puskesmas, and pharmacies compared to rural areas (p < 0.001). However, rural areas have a greater density of nurses than urban areas (p < 0.001). There was no significant difference in *Posbindu* density between urban and rural areas (p = 0.054).

Table 2 outlines the clinical and lifestyle characteristics of the individuals by sex and rurality. The average SBP and DBP of the respondents were 131.7 (SD = 22.0) mmHg and 80.9 (11.0), respectively. The mean values for SBP, DBP, and BMI were significantly higher for males (p < 0.001) and urban residents (p < 0.001) compared to females and rural residents. Females (p < 0.001) and rural residents (p < 0.001) had a higher mean random plasma glucose level than males and urban residents. Approximately one in ten individuals were smokers, with the majority being males (p < 0.001). A higher proportion of females (p < 0.001) and rural residents (p < 0.001) reported high salt intake (>1 tablespoon/day) compared to males and urban residents. A lower proportion of females (p < 0.001) and rural residents (p < 0.001) reported consuming fruits and vegetables less than 5 portions/day compared to males and urban residents. Approximately 42.7% of individuals reported doing physical activity less than 3 times/week, with a higher proportion being males (p < 0.001) and urban residents (p < 0.001). We further found that nearly one in five individuals aged 40 and above had a high 10-year risk of CVD, with a significantly higher proportion among urban (19.6%) than rural (18.3%) residents (p < 0.001).

**Table 2:** Clinical and lifestyle characteristics of individuals based on sex and rurality.

Variables	All (n = 903,130)	Female (n = 518,922)	Male (n = 384,208)	p-value	Urban (n = 367,151)	Rural (n = 535,979)	p-value	Missing data**
Mean systolic blood pressure (mmHg), mean (SD)	131.7 (22.0)	132.5 (22.6)	130.7 (21.2)	<0.001	132.4 (22.1)	131.3 (22.0)	<0.001	0 (0)
Mean diastolic blood pressure (mmHg), mean (SD)	80.9 (11.0)	81.1 (11.2)	80.6 (10.7)	<0.001	81.4 (11.2)	80.6 (10.8)	<0.001	0 (0)
Body Mass Index (kg/m2), mean (SD)	25.0 (4.2)	25.6 (4.3)	24.2 (4.0)	<0.001	25.0 (4.1)	24.9 (4.3)	<0.001	0 (0)
Random plasma glucose (mg/dL), mean (SD)	151.5 (49.5)	153.1 (52.5)	149.3 (4.3)	<0.001	148.7 (48.6)	153.6 (50.0)	<0.001	700,076 (77.52%)
Smoking, frequency (%)	91,284 (10.1)	11,583 (2.0)	79,701 (20.8)	<0.001	39,529 (10.8)	51,755 (9.7)	<0.001	661 (0.07%)
High intake of salt (>1 tablespoon/day), frequency (%)	197,577 (21.9)	117,530 (22.7)	80,047 (20.8)	<0.001	79,151 (21.6)	118,426 (22.1)	<0.001	622 (0.07%)
Have physical activity less than 3 times/week, frequency (%)	385,328 (42.7)	210,861 (40.6)	174,467 (45.4)	<0.001	159,846 (43.5)	225,482 (42.1)	<0.001	116 (0.01%)
Consume fruits and/or vegetables <5 portions/day, frequency (%)	104,556 (11.6)	46,385 (11.2)	58,171 (12.1)	<0.001	37,270 (10.2)	67,286 (12.6)	<0.001	0 (0)
Ever diagnosed with diabetes mellitus, frequency (%)	21,000 (2.3)	14,308 (2.8)	6,692 (1.7)	<0.001	9,723 (2.7)	11,277 (2.1)	<0.001	661 (0.07%)
Ever diagnosed with hypertension, frequency (%)	80,443 (8.9)	54,468 (10.5)	25,975 (6.8)	<0.001	33,815 (9.2)	46,628 (8.7)	<0.001	661 (0.07%)
Ever diagnosed with cardiovascular disease	12,678 (1.5)	6,146 (1.2)	6,532 (1.7)		5,541 (1.3)	7,137 (1.4)		661 (0.07%)
10-year risk of cardiovascular disease, frequency (%)								146 (0.02%)
<5%	411,669 (45.6)	249,393 (48.1)	162,276 (42.3)	<0.001	170,013 (46.3)	241,656 (45.1)	<0.001	
5 to <10%	260,775 (29.8)	167,430 (32.2)	93,345 (24.3)		108,480 (29.6)	152,295 (28.4)		
10% to <20%	194,786 (21.6)	95,620 (18.4)	99,166 (25.8)		75,104 (20.5)	119,682 (22.3)		
20% to <30%	27,767 (3.1)	5,921 (1.1)	21,846 (5.7)		10,683 (2.9)	17,084 (3.2)		
>=30%	7,472 (0.8)	139 (0.0)	7,333 (1.9)		2,548 (0.7)	4,924 (0.9)		
High cardiovascular disease risk, frequency (%)								146 (0.02%)
Yes	169,758 (18.8)	72,181 (18.7)	97,577 (18.8)	0.839	71,779 (19.6)	97,979 (18.3)	<0.001	
No	733,226 (81.2)	311,966 (82.3)	421,260 (81.2)		295,271 (80.4)	437,955 (81.7)		
Year cardiovascular disease risk screening, frequency (%)								0 (0)
January – December 2020	217,946 (24.1)	128,559 (24.8)	89,387 (23.3)	<0.001	91,574 (24.9)	126,372 (23.6)	<0.001	
January – December 2021	168,045 (18.6)	94,061 (18.1)	73,984 (19.3)		65,746 (17.9)	102,299 (19.1)		
January – December 2022	211,433 (23.4)	120,370 (23.2)	91,063 (23.7)		85,038 (23.2)	126,395 (23.6)		
January – December 2023	271,544 (30.1)	156,209 (30.1)	115,335 (30.0)		110,272 (30.0)	161,272 (30.1)		
January – February 2024	34,162 (3.8)	19,723 (3.8)	14,439 (3.8)		14,521 (4.0)	19,641 (3.7)		

SD = standard deviation. * Univariable analyses using Kruskal-Wallis test for continuous variables and chi-square tests for categorical variables.** Presented are n (%)

#### Prevalence of cardiovascular disease risk factors and high 10-year risk stratification based on sex and rurality.

**Fig 2:**
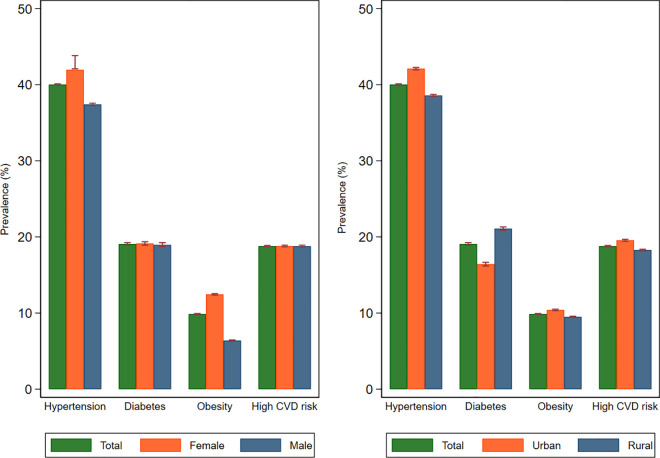
Prevalence of cardiovascular disease risk factors and high 10-year risk stratification based on sex and rurality in Malang district in Indonesia.

Fig 2 illustrates the prevalence of CVD risk factors, including hypertension, diabetes, and obesity, as well as the estimated 10-year CVD risk, categorized by sex and rurality. Hypertension emerged as the most prevalent risk factor at 40.0%, followed by diabetes at 16.1%, and obesity at 9.9%. The data indicated that hypertension was more common among females and in urban areas. Similarly, obesity rates were higher in females compared to males. The overall prevalence of high 10-year CVD risk was 18.8%, with urban areas showing a higher prevalence (19.5%, 95% CIs 19.4–19.7%) compared to rural areas (18.8%, 95% CIs 18.2–18.4%).

### Multilevel logistic regression analyses

The results of the multilevel logistic regression analyses are presented in Table 3. The univariable analyses in Model 1 show that individuals in rural areas had lower odds of having a high estimated 10-year CVD risk than those in urban areas (COR = 0.920; 95% CI = 0.910–0.930). Those who were divorced (COR = 2.965; 95% CI = 2.897–3.036) and widowed (COR = 2.152; 95% CI = 2.111–2.196) were more likely to have high CVD risk than those who were married. Single individuals were less likely to have high CVD risk than married individuals (COR = 0.532; 95% CI = 0.520–0.545). These relationships remain when all the risk factors are included in Models 2 and 3. In the final model, rural residents were less likely than urban residents to have a high estimated 10-year CVD risk (AOR = 0.844; 95% CI = 0.747–0.954). Compared to married individuals, those who were divorced (AOR = 1.722; 95% CI = 1.675–1.772) and widowed (AOR = 1.386; 95% CI = 1.352–1.421) were more likely to have a high estimated 10-year CVD risk. Compared to married individuals, single individuals had a lower likelihood of having a high CVD risk (AOR = 0.0.732; 95% CI = 0.712–0.754).

**Table 3:** Socio-demographic and healthcare access determinants of high estimated 10-year cardiovascular risk.

Variables	Model 1		Model 2		Model 3	
	Crude Odds Ratio	95% CI	Adjusted Odds Ratio	95% CI	Adjusted Odds Ratio	95% CI
*Individual-level variables*						
Marital status						
Married	**1.000**		**1.000**		**1.000**	
Single	0.532***	0.520 - 0.545	0.737***	0.717 - 0.758	0.732***	0.712 - 0.754
Divorced	2.965***	2.897 - 3.036	1.793***	1.745 - 1.842	1.722***	1.675 - 1.772
Widowed	2.152***	2.111 - 2.196	1.433***	1.399 - 1.469	1.386***	1.352 - 1.421
Education level						
Not attending school	**1.000**		**1.000**		**1.000**	
Elementary school	1.889***	1.843 - 1.935	1.237***	1.201 - 1.273	1.159***	1.124 - 1.194
Junior secondary school	1.668***	1.629 - 1.708	1.154**	1.120 - 1.189	1.124***	1.090 - 1.159
High school	0.821***	0.796 - 0.847	0.808***	0.779 - 0.837	0.777***	0.749 - 0.852
University	0.791***	0.758 - 0.826	0.823***	0.781 - 0.866	0.809***	0.768 - 0.852
Employment status						
Unemployed	**1.000**		**1.000**		**1.000**	
Formal	0.742***	0.718 - 0.766	0.762***	0.734 - 0.791	0.760***	0.731 - 0.790
Informal	1.867***	1.824 - 1.911	1.239***	1.107 - 1.172	1.189***	1.154 - 1.225
Homemaker	1.036	1.009 - 1.065	0.914***	0.886 - 0.942	0.926***	0.896 - 0.956
Retired	1.827***	1.726 - 1.933	1.798***	1.690 - 1.914	1.798***	1.688 - 1.916
Self-employed	0.997***	0.965 - 1.031	0.835***	0.804 - 0.866	0.815***	0.785 - 0.847
High intake of salt (> tablespoon/day)	1.044***	1.030 - 1.057	1.705***	1.681 - 1.730	1.740***	1.714 - 1.766
Have physical activity <3 times/week	2.743***	2.713 - 2.774	2.464***	2.434 - 2.494	2.369***	2.340 - 2.399
Consume fruits and/or vegetables <5 portions/day	1.711***	1.685 - 1.736	1.585***	1.558 - 1.613	1.674***	1.644 - 1.705
Have BPJS insurance	0.327***	0.323 - 0.331	0.367***	0.363 - 0.371	0.389***	0.384 - 0.394
Living in rural areas	0.920***	0.910 - 0.930	0.888***	0.876 - 0.898	0.844**	0.747 - 0.954
Year CVD risk screening						
January – December 2020	**1.000**		**1.000**		**1.000**	
January – December 2021	1.242***	1.219 - 1.265	0.988	0.969 - 1.007	1.050***	1.029 - 1.070
January – December 2022	1.336***	1.314 - 1.359	1.049***	1.031 - 1.068	1.041***	1.021 - 1.060
January – December 2023	2.392***	2.356 - 2.258	1.715***	1.687 - 1.743	1.591***	1.564 - 1.618
January – February 2024	2.197***	2.137-2.258	1.484	1.441-1.528	1.362***	1.321-1.405
*Village-level variables*						
Medical doctor per 1000 population	0.834***	0.812- 0.857			0.819	0.652 - 1.030
Posbindu per 1000 population 40+	0.982***	0.980 - 0.984			0.981**	0.966 - 0.996
Pharmacy per 1000 population	0.922***	0.883- 0.962			1.092	0.702 - 1.697
Nurses per 1000 population	0.997***	0.996 - 0.997			0.994**	0.988 - 0.999
Population (log)	0.891***	0.884 - 0.899			0.785***	0.713 - 0.865
Access to nearest Puskesmas	0.930***	0.920 - 0.941			0.941	0.852 - 0.038
Constant			0.142***	0.137 - 0.147	2.346	0.891- 6.178
Level 1 variance (individual)					0.471	0.438 - 0.507
Level 2 variance (village)					0.216	0.187 - 0.250
−2 Loglikehood			−386286.91		−374703.54	
ICC					0.062	
Number of individuals	902,469		902,469		906,469	
Number of villages	390		390		390	

*** p < 0.01, ** p < 0.05; Model 1 = Model 2 = Model 3=

Focusing on socioeconomic risk factors, the univariable analyses in Model 1 show that individuals educated in elementary (COR = 1.889; 95% CI = 1.843–1.935) and junior high schools (COR = 1.668; 95% CI = 1.629–1.708) were more likely to have high CVD risk than those who did not attend school. Those who were educated at high school (COR = 0.821; 95% CI = 0.796–0.847) and university (COR = 0.791; 95% CI = 0.758–0.826) were less likely to have high CVD risk than those who did not attend school. Informal workers (COR = 1.867; 95% CI = 1.824–1.911) and retired (COR = 1.827; 95% CI = 1.726–1.933) were more likely to have high CVD risk than unemployed individuals. Formal (COR = 0.742; 95% CI = 0.718–0.766) and self-employed (COR = 0.997; 95% CI = 0.965–1.031) were less likely to have high CVD risk than unemployed individuals. Individuals who reported an intake of salt more than recommended (COR = 1.044; 95% CI = 1.030–1.057), had physical activity less than 3 times per week (COR = 2.743; 95% CI = 2.713–2.774), and consumed vegetables or fruit less than 5 portions/day had greater CVD risk (COR = 1.711; 95% CI = 1.685–1.736). BPJS members were less likely to have a high risk of CVD than non-BPJS members (COR = 0.327; 95% CI = 0.323–0.331). The odds of people with high CVD risk are higher in 2021–2024 than in 2020.

These associations remain when Models 2 and 3 incorporate all risk factors in the multivariable analysis. The final model shows those who attended elementary (AOR = 1.159; 95% CI = 1.124–1.194) and junior high (AOR = 1.124; 95% CI = 1.090–1.159) schools were shown to have a higher risk of CVD than people who did not go to school. High school graduates (AOR = 0.777; 95% CI = 0.749–0.852) and university graduates (AOR = 0.809; 95% CI = 0.768–0.852) were less likely than non-graduates to be at high risk for CVD. Compared to unemployed people, informal workers (AOR = 1.189; 95% CI = 1.154–1.225) and retirees (AOR = 1.798; 95% CI = 1.688–1.916) were more likely to have a high CVD risk. Self-employed people (AOR = 0.815; 95% CI = 0.785–0.847) and formal workers (AOR = 0.760; 95% CI = 0.731–0.790) were less likely than unemployed people to have a high CVD risk.

The findings from Model 3 indicate that the density of pharmacies (AOR = 1.092; 95% CI = 0.702–1.697) and medical doctors (AOR = 0.819; 95% CI = 0.652–1.030) did not significantly affect the likelihood of having a high estimated 10-year CVD risk. Conversely, individuals residing in villages with higher densities of *Posbindu* (AOR = 0.981; 95% CI = 0.731–0.790) and nurses (AOR = 0.994; 95% CI = 0.988–0.999) were less likely to have a high 10-year CVD risk. Additionally, those living in villages with an easy access to Puskesmas had a lower likelihood of high estimated 10-year CVD risk compared to those in villages with poorer access, although this association was not statistically significant.

The variance in high estimated 10-year CVD risk at the individual level was 47.1%, indicating that 52.9% of the variance is attributable to higher-level factors. This suggests that determinants beyond individual characteristics may influence the variation in high estimated 10-year CVD risk. The intra-class correlation was 6.2%, meaning that 6.2% of the variation in high 10-year cardiovascular risk occurred at the village level. Additionally, the −2 Loglikelihood value indicates a substantial improvement in model fit for Model 3 compared to Model 2.

### Treatment of hypertension and CVD risk based on sex and rurality

Table 4 presents preventive treatments among individuals with hypertension, diabetes, and high CVD risk based on sex and rurality. Of all the respondents with a high estimated 10-year CVD risk (n = 169,758), 57,421 and 53,693 were on blood pressure lowering and statin treatment, respectively. The proportion of females with an estimated 10-year CVD risk receiving blood pressure lowering treatment was higher than males, but not for statin and optimal treatment. Only 43,557 received optimal preventive treatment. However, the use of acetylsalicylic acid (ASA), including aspirin, for primary prevention of CVD in high-risk individuals without established CVD remains somewhat uncertain. The proportion of individuals with an estimated 10-year CVD risk in urban areas with optimal preventive treatments (29%) was higher than those in rural areas (22%).

**Table 4:** Preventive treatments among individuals with high CVD risk based on sex and location.

		On blood pressure lowering treatment	On statin treatment	On optimal preventive treatment
Total	n/N	57,421/169,758	53,693/169,758	43,557/169,557
	%	33.83	31.62	25.68
	CI	33.60-34.05	31.40-31.85	25.45-25.86
Female	n/N	32,769/97,577	30,421/53,693	23,693/97,577
	%	35.58	31.17	24.28
	CI	33.28-33.88	30.88-31.46	24.01-24.55
Male	n/N	24,653/72,181	23,272/72,181	19,864/72,181
	%	34.15	32.24	27.51
	CI	33.80-34.50	31.90-32.58	27.19-27.84
*P*-value		≤ 0.050	≤ 0.001	≤ 0.001
Urban	n/N	26,104/71,779	24,747/71,779	21,198/71,779
	%	36.36	34.47	29.53
	CI	36.01-36.72	34.12-34.82	29.19-29.86
Rural	n/N	31,317/97,979	28,946/97,979	22,358/97,979
	%	31.96	29.54	22.81
	CI	31.67-32.25	29.25-29.83	22.55-23.08
*P*-value		≤ 0.001	≤ 0.001	≤ 0.001

## Discussion

This study is the first large, cross-sectional, population-based study to address the prevalence of the estimated 10-year risk of CVD using the most recent WHO/ISH scoring system in Indonesia. We found that almost two out of ten adults aged 40 years and older have a high estimated 10-year CVD risk. This prevalence was lower compared to the previous study of eight villages in the same district, which reported that almost 30% of the adults in the same age group had a high CVD risk [5]. This current study included all 390 villages in the Malang district, providing a more complete picture of CVD risk prevalence in the region. With respect to other studies that used the WHO/ISH risk scores, the prevalence of high 10-year cardiovascular risk that we found in Indonesia is higher than that of rural India (10.2%) [24] and those in Cambodia (10.4%) with the same age range of sample (40 years and older. However, the prevalence was lower compared to the prevalence of high CVD risk in Mongolia (33.3%) and Malaysia (20.8%) [25].

The prevalence of high estimated 10-year CVD risk was slightly higher among male and among residents of urban villages than female and those live in rural villages. While there is a statistically significant difference in these prevalence rates, the absolute differences between individuals with high estimated 10-year CVD risk based on rurality are quite large. A higher prevalence of high CVD risk among urban populations than their rural counterparts has been previously observed worldwide [26]. Our findings suggest that increasing life expectancy and urbanization are major determinants of CVD in developing countries. Urbanization has been associated with higher CVD incidence [27], including in Southeast Asian countries [28,29]. Over half of Indonesians (57.3%) were living in urban areas in 2021, and this proportion was projected to increase to 66% by 2035 [30].

In addition to nutritional issues, physical activities and tobacco smoking are important risk factors for cardiovascular diseases. Our study found that 10.3% of adults 40 and older are active smokers with majority of them are male individual. On the other hand, more female individuals are facing lifestyle issues, i.e., obesity, insufficient physical activity, less fruit and vegetable consumption, and high intake of salt. Our finding that a high salt intake was related to high estimated 10-year CVD risk corroborated prior studies [31,32]. Excessive sodium consumption, which the World Health Organisation defines as more than 5 grams per day, has been found to raise blood pressure significantly and has been associated with the development of CVDs [33]. The findings of this study on the significant association between less vegetables and/or fruits intake and higher estimated 10-year CVD risks support the literature. A recent meta-analysis revealed that increased consumption of fruit and vegetables prevents the risk of developing hypertension [34]. Our findings further confirm prior studies that reported urbanization in Indonesia is associated with dietary patterns conducive to increase Non-Communicable Disease risk-for example, high in soft drinks and ultra-processed foods, and consumption of local traditional foods is resilient to the effect of urbanization [30].

Our study shows several important findings regarding the treatment received by the respondents with high cardiovascular risk in Indonesia. We found that only a quarter of respondents with a high estimated 10-year CVD risk were on optimal preventive treatment, with males and those who live in urban areas reported having better treatment than females and those who live in rural areas. This findings support prior study on unmet need for CVD care in Indonesia, which found that residence in urban areas was significantly associated with met needs for CVD care [6]. These findings show the urgent need for the primary healthcare system to strengthen and address this need. These findings reinforce the evidence that, notwithstanding the efforts of the government to increase healthcare access, inequality based on sex and rurality persists where cardiovascular care is concerned.

As cardiovascular risk is largely asymptomatic, the capacity of the health system to provide information and diagnostic services to the population is crucial for awareness of cardiovascular risk. The findings of multilevel analyses highlight the variation of high estimated 10-year cardiovascular risk factors beyond individual characteristics related to the health system’s capacity. In this research, we found that the higher density of community-based healthcare (*Posbindu*) was related to lower cardiovascular risk. These findings suggest the potential of community-based early detection of cardiovascular risk at the community or grassroots level [35]. The ratio of medical doctor and nurses per 1000 population were related to less high cardiovascular risk. Moreover, individuals covered by BPJS insurance were less likely to have high CVD risk than those not covered by the national insurance. These findings confirm the importance of the availability of financial support and accessibility of preventive care in the management of CVD risk prevention [36–38]. The null finding of the association of pharmacy per 1000 population on high cardiovascular risk also indicates a lack of access to treatment, especially for those with high CVD risk. Prior studies have also reported the lack of access to treatment among people with CVD risk in Indonesia [6,39].

This study has a number of limitations. Firstly, some of our data were based on self-reported information. Although the interviews were carried out by well-trained personnel, the potential for bias was present (e.g., over- or underdiagnoses due to recall issues or subjectivity in reporting symptoms). Another limitation is that this research was based on residents in the district of Malang only, which might not represent the wider population in other districts and provinces in Indonesia. Finally, a significant proportion (77.5%) of participants had missing random blood glucose data, which restricted our ability to use laboratory-based CVD risk prediction methods. To address this, we relied on the WHO/ISH non-laboratory-based risk prediction charts, which have been specifically designed for use in resource-limited settings where biomarker data may be incomplete or unavailable. While this approach ensures consistency in risk estimation and avoids selection bias, it may lead to an underestimation of CVD risk among individuals with undiagnosed diabetes, as blood glucose levels were not incorporated into the risk assessment.

Despite these limitations, we have demonstrated a number of implications that are useful for the literature and policy-makers. It used large data that is representative of the whole population. The third limitation is that the analysis used cross-sectional data, limiting this study to establishing cause-and-effect relationships. Finally, it used standardized data collection tools. For example, the interviews and physical examinations were performed by trained enumerators. The way they measured height, weight and blood pressure follows a standard protocol [14,15]. This study shows that the high estimated 10-year CVD risk in Malang district is not only associated with individual socioeconomic status and lifestyle but also strongly associated with village health systems’ capacity.

From an empirical perspective, this study suggests that it is essential to consider a range of social and health system determinants beyond individual factors and examine their effects on high cardiovascular risks. Several public health interventions can be considered in improving CVD health. First, implement public health programs aimed at raising awareness about the importance of preventive treatment for cardiovascular diseases and promoting early intervention strategies. This could include community-based education campaigns and initiatives to encourage regular health screenings focusing on female and rural areas. Second, foster collaboration and integration between primary care providers, specialists, and community health workers to ensure a coordinated approach to delivering preventive treatments for cardiovascular diseases. This could involve establishing referral pathways and multidisciplinary care teams to streamline the delivery of care. Third, embrace telemedicine and remote monitoring technologies to overcome geographical barriers and improve access to treatments for individuals living in remote or rural areas. This could include virtual consultations, remote medication management, and telehealth programs focused on chronic disease management. Fourth, implementing policies that support healthy lifestyles, such as increasing taxes on tobacco products, regulating salt content in processed foods, and promoting urban planning that encourages physical activity, can create environments conducive to cardiovascular health.

## Supporting information

S1 Table Deficit variables included in the ELSA frailty index. (DOCX)
