# Supplementary material for: Healthcare access and socio-demographic determinants of estimated 10-year risk of cardiovascular diseases in Indonesia: A population-based study
Source: PLoS One. 2025 Aug 20;20(8):e0318112. doi: 10.1371/journal.pone.0318112 (PMC12367158; doi:10.1371/journal.pone.0318112)
Supplement: S1 Table — (DOCX) [file pone.0318112.s001.docx]

Supplementary S1: Variables and definition used in the study

| Variables | Definition |
| --- | --- |
| Age (years) | Age of respondents in years. Age was classified into: 40-44, 45-49, 50-54, 55-59, 60-64, 65-69, and 70+. |
| Education level | The highest education level of respondent. Education level was classified into: Not attending school, elementary school, junior secondary school, high school, and university. |
| Marital status | Respondents’ marriage status: married, single, divorced, and widowed. |
| Employment status | Current status of respondents’ employment. Employment status was classified into: unemployed, career, casual, housewife, retired, self-employed. |
| Have BPJS insurance | Respondents subscribe BPJS insurance (1 = Yes, 0 = No) |
| Medical doctor per 1000 population | Total number of doctors per 1000 village population. |
| *Posbindu* per 1000 population 40+ | Total number of *Posbindu* per 1000 village population 40 and older |
| Pharmacy per 1000 population | Total number of registered pharmacies per 1000 village population |
| Nurses per 1000 population | Total number of nurses per 1000 village population |
| Access to nearest *Puskesmas* (accessible) | Subjective evaluation of access to nearest *Puskesmas* which classified into not accessible (0) and accessible (1). |
| Population 40 and older | Total village population 40 and older |
| Population | Total village population for any ages |
| Mean systolic BP (mmHg) | Mean of systolic blood pressure (mmHg) |
| Mean diastolic BP (mmHg) | Mean of diastolic blood pressure (mmHg) |
| BMI (kg/m2) | Body mass indexes (kg/m2) |
| Random plasma glucose (mg/dL) | Respondents blood glucose levels at any given point in the day (mg/dL) |
| Smoking | Respondents is currently smoking which classified into smoking (1) and no smoking/previously smoking (0). |
| High intake of salt | Respondents reported consume salt more than 1 table spoons every day which classified into high intake salt (1) and otherwise (0) |
| Low levels of physical activity | Respondents reported having moderate physical activity less than 3 time a week (1 = Yes, 0 = No) |
| Less intake of fruits and/or vegetables | Respondents reported consume less than five portion of vegetables/fruits everyday (1=Yes, 0=No) |
| Ever diagnosed with diabetes mellitus | Respondents reported ever diagnosed with diabetes mellitus by doctor (1=Yes, 0=No) |
| Ever diagnosed with hypertension | Respondents reported ever diagnosed with hypertension by doctor (1=Yes, 0=No) |
| 10-year risk of CVD | The 10-year risk of CVD using the World Health Organization cardiovascular disease (WHO CVD) risk (non-laboratory-based) charts calibrated for use in Southeast Asia region B (SEAR B) |
| Ever diagnosed by CVD | Respondents reported ever diagnosed with CVD by doctor (1=Yes, 0=No) |
| Clinically high-risk | systolic blood pressure >160 mm Hg or diastolic blood pressure >100mmHg (1=Yes, 0=No) |
| High CVD risk | The presence of any of the following: (1) a history of CVD confirmed by a physician, (2) an extreme blood pressure elevation (systolic blood pressure ≥160 mmHg or diastolic blood pressure ≥100mmHg, (3) a 10-year estimated CVD risk of 20% or more, or (4) a 10-year estimated CVD risk < 20% and systolic blood pressure ≥140 mmHg and/or diastolic blood pressure ≥90mmHg (1= Yes, 0=No). |
| Year CVD risk screening | Years when CVD risk screening was first time conducted. |
